# Long-term survival after transcatheter aortic valve implantation in nonagenarian patients: a multicenter real-world study

**DOI:** 10.3389/fcvm.2026.1945453

**Published:** 2026-10-08

**Authors:** Gianluca Pagnoni, Marco Loffi, Dalia Caleffi, Susan Darroudi, Giuseppe Masaracchia, Benedetta Frassoni, Francesco Marangi, Sophia di Michele, Davide Gabbieri, Luciano Losi, Alberto Monello, Fabio Alfredo Sgura, Salvatore Arrotti, Rosario Rossi, Daniele Iaccarino, Marco Ruozzi, Vincenzo Guiducci, Filippo Luca Gurgoglione, Giorgio Benatti, Luigi Vignali, Giampaolo Niccoli, Giuseppe Boriani, Daniela Aschieri, Francesca Coppi

**Affiliations:** 1 Cardiology Unit, Specialistic Medicine Department, Guglielmo da Saliceto Hospital, Piacenza, Italy; 2 National Institute for Cardiovascular Research (INRC), Bologna, Italy; 3 Division of Cardiology, Azienda Socio-Sanitaria Territoriale di Cremona, Cremona, Italy; 4 Department of Biomedical, Metabolic and Neural Sciences, University of Modena and Reggio Emilia, Modena, Italy; 5 Department of Medical and Surgical Sciences for Children and Adults, University of Modena and Reggio Emilia, Modena, Italy; 6 Cardiology Division, Department of Biomedical Metabolic and Neural Sciences, University of Modena and Reggio Emilia, Modena, Italy; 7 Cardiology Department, Azienda Ospedaliero-Universitaria of Parma, Parma, Italy; 8 Cardiac Surgery Division, Hesperia Hospital, Modena, Italy; 9 Cardiology Department, Nuovo Ospedale S. Agostino Estense, Modena, Italy; 10 Cardiology Unit, Azienda USL-IRCCS di Reggio Emilia, Reggio Emilia, Italy

**Keywords:** long-term survival, mortality, nonagenarians, procedural outcomestranscatheter aortic valve implantation (TAVI), severe aortic stenosis, survival, transcatheter aortic valve implantation

## Abstract

**Background:**

The growing number of nonagenarian patients with severe aortic stenosis has increased interest in transcatheter aortic valve implantation (TAVI) in this population. However, data on long-term outcomes remain limited. This study aimed to evaluate long-term survival and procedural outcomes of TAVI in nonagenarians and to explore clinical factors associated with long-term mortality.

**Methods:**

We conducted an ambispective multicenter observational cohort study including patients aged ≥90 years who underwent TAVI for severe aortic stenosis between 2013 and 2021 at six centers in the Emilia-Romagna region, Italy. Baseline and procedural data were collected retrospectively, while outcome ascertainment combined prospective follow-up contacts with retrospective verification of dates of death or last confirmed vital status. Survival was estimated using the Kaplan–Meier method, and associations with mortality were examined using an exploratory Cox regression analysis.

**Results:**

A total of 60 nonagenarian patients (mean age 91 ± 1.5 years; 67% female) were included. Procedural success was achieved in 97% of cases. In-hospital mortality was 5%, and 30-day mortality was 8.3%. Over a mean observation period of 5.7 years from TAVI to death or the last confirmed vital-status assessment, the Kaplan–Meier estimated 5-year survival was 35%. In the exploratory multivariable analysis, male sex and chronic obstructive pulmonary disease (COPD) were associated with higher long-term mortality.

**Conclusions:**

In patients aged ≥90 years selected by the Heart Team for TAVI, the Kaplan–Meier estimated 5-year survival was 35%. The procedure demonstrated a high success rate and an acceptable safety profile. Male sex and COPD showed an exploratory association with worse prognosis. These findings suggest that TAVI may be a feasible option in carefully selected nonagenarians when guided by comprehensive clinical assessment and individualized decision-making.

## Introduction

Life expectancy in developed countries is steadily increasing. In Italy, life expectancy at birth was estimated at 83.4 years in 2024 (1). As a result, the number of nonagenarian patients with symptomatic severe aortic stenosis, a potentially fatal condition if untreated, is growing. Patients of such advanced age are, in most cases, excluded from surgical aortic valve replacement because of high operative risk. The introduction of transcatheter aortic valve implantation (TAVI) has revolutionized the treatment of aortic stenosis in high-risk or inoperable patients, making intervention feasible even at very advanced ages (2, 3). However, nonagenarians generally have a greater burden of comorbidities and frailty, translating into higher procedural risk and mortality than in younger patients (4, 5). According to data from the US Transcatheter Valve Therapy (TVT) Registry, approximately 16% of patients undergoing TAVI are aged ≥90 years, and in this group 30-day mortality (9% vs. 6%) and 1-year mortality (25% vs. 22%) are modestly higher than in patients aged <90 years (6). Nevertheless, several clinical series have suggested that TAVI is feasible in carefully selected nonagenarians, with acceptable early outcomes and improved quality of life among survivors (6–8). For example, a recent study of 75 nonagenarians treated with TAVI found no significant difference in 1-year mortality compared with octogenarian patients (21.3% vs. 16.7%, *p* = 0.405) (9). By contrast, meta-analyses including large cohorts have shown that age ≥90 years is associated with an increase in both early and mid-term mortality after TAVI (Hazard Ratio (HR) approximately 1.3–1.5 vs. patients aged <90 years) (10). Overall, the available evidence conveys a dual message: on the one hand, TAVI in nonagenarians may provide substantial clinical benefit; on the other hand, careful candidate selection is essential to avoid futile procedures in patients with a very limited life expectancy.

In light of these considerations, the present study was designed to evaluate the long-term outcomes of nonagenarian patients undergoing TAVI in a regional real-world setting and to explore the main variables associated with mortality. The primary endpoint was 5-year survival after the procedure. Secondary endpoints were procedural success, periprocedural safety, and the exploratory identification of clinical factors associated with mortality during follow-up. The study hypothesis was that, in patients carefully selected by the Heart Team, TAVI could provide clinically meaningful long-term survival.

## Materials and methods

### Study design and population

An ambispective multicenter observational cohort study was conducted. Through the Emilia-Romagna North TAVI registry, including Ospedale Civile di Piacenza, Ospedale Civile Sant’Agostino-Estense, Policlinico di Modena, Hesperia Hospital Modena, Ospedale Maggiore di Parma, and Arcispedale Santa Maria Nuova, all patients aged ≥90 years who underwent transcatheter aortic valve implantation between January 2013 and December 2021 were retrospectively identified. Outcome ascertainment combined retrospective and prospective components. For patients who were alive when prospective follow-up was initiated, subsequent information was obtained through outpatient visits or telephone contacts. When a patient had already died before prospective contact, vital status and the date of death were retrospectively ascertained from medical records and municipal registry records. Inclusion criteria were age ≥90 years at the time of the procedure, symptomatic severe aortic stenosis confirmed by echocardiography, and a favorable indication for the procedure by the local Heart Team in accordance with international guidelines. Candidate selection was performed independently by the local Heart Team at each participating center according to contemporaneous clinical practice. Guideline recommendations advise against intervention when severe comorbidities make improvement in quality of life or survival beyond 1 year unlikely (11). However, the registry documented only the final Heart Team indication and did not include a harmonized cross-center selection protocol or a standardized patient-level record of the criteria underlying that decision. Consequently, the present dataset cannot determine whether candidate-selection thresholds were applied consistently across centers. Patients with non-severe aortic stenosis, those undergoing bail-out TAVI during cardiac surgery, and those undergoing repeat intervention on a bioprosthetic valve (valve-in-valve) were excluded. All patients provided informed consent for data collection. The study protocol was approved by the AVEN Ethics Committee (Area Vasta Emilia Nord; study code 726/2018) on 4 January 2019, and institutional authorization was subsequently obtained on 8 January 2019.

The study was reported in accordance with the Strengthening the Reporting of Observational Studies in Epidemiology (STROBE) statement (12).

### TAVI procedure and endpoint definitions

TAVI procedures were performed according to each center's standard practice. Periprocedural complications were classified according to the Valve Academic Research Consortium-2 (VARC-2) criteria, where applicable. For the purposes of the present analysis, procedural success was defined using a study-specific definition comprising successful valve implantation, a residual transvalvular gradient <20 mmHg, and the absence of major intraprocedural complications. Because the registry did not systematically capture all components required for the formal VARC-2 device-success endpoint, the two definitions should not be considered equivalent.

In particular, the composite early safety endpoint (at 30 days) was defined as the occurrence of at least one of the following complications: acute kidney injury (AKI), a cerebrovascular event within 30 days (stroke/TIA (Transient Ischemic Attack)/RIND (Reversible Ischemic Neurological Deficit)), major bleeding, major vascular complication, postprocedural pacemaker implantation, or cardiac tamponade.

### Clinical and echocardiographic variables

Baseline clinical characteristics were collected for each patient, including demographic data (age and sex), body mass index, major comorbidities (hypertension, dyslipidaemia, diabetes mellitus, chronic obstructive pulmonary disease [COPD], chronic kidney disease stage ≥3, peripheral artery disease, atrial fibrillation, and previous ischemic heart disease), and surgical risk scores. Preprocedural echocardiographic parameters were also recorded, including aortic valve area, mean transaortic gradient, left ventricular ejection fraction (LVEF), and the presence of other valvular heart diseases. In particular, mitral regurgitation (MR) severity was classified according to echocardiographic guidelines. The presence of concomitant aortic regurgitation was also assessed.

Postprocedural echocardiographic assessment was not governed by a uniform study protocol. The registry captured the residual transvalvular gradient required for the definition of procedural success and the presence of moderate-to-severe postprocedural aortic regurgitation. Other quantitative postprocedural measurements and serial echocardiographic follow-up were not systematically available across centers and were therefore not analyzed.

Validated measures of frailty, functional status, cognition, nutritional status, activities of daily living, and estimated life expectancy were not included in the analysis because these variables were not systematically recorded across all centers and study years.

### Follow-up and outcomes

Clinical follow-up, when available, consisted of routine outpatient cardiology visits or telephone contacts at 1 month, 6 months, 1 year, and annually thereafter. Outcome ascertainment combined these prospective contacts with retrospective review of medical and municipal registry records. For patients who had died before prospective contact, the date of death was obtained retrospectively; patients remaining alive were censored at the date of their last confirmed vital-status assessment. Because vital-status information was updated at different times across the participating centers, the registry did not apply a single common administrative censoring date. Observation time was defined as the interval from TAVI to all-cause death or the last confirmed alive assessment. Completeness of survival follow-up was assessed by verifying the availability of either a date of death or a date of last confirmed alive assessment for each patient. Standardized longitudinal measures of NYHA functional class, frailty, exercise capacity, independence in activities of daily living, and quality of life were not systematically collected across all centers and time periods. Complete and consistently adjudicated causes of long-term death were not systematically available. Consequently, cardiovascular and non-cardiovascular mortality could not be analyzed reliably, and the longitudinal outcome analysis was limited to all-cause survival.

The primary endpoint was the Kaplan–Meier estimated probability of all-cause survival at 5 years after TAVI. Secondary endpoints included procedural success, periprocedural safety, and the exploration of clinical and procedural variables associated with long-term mortality.

## Statistical analysis

Continuous variables were described as mean ± standard deviation (SD) if normally distributed, and as median and IQR (interquartile range) otherwise. Categorical variables were expressed as absolute numbers and percentages. Comparisons between categorical variables were performed using chi-square or Fisher's exact test. Comparisons between continuous variables were performed using the independent-samples t-test for normally distributed variables and the Mann–Whitney U test for non-normally distributed variables. Overall survival was calculated from the date of TAVI to the date of all-cause death or the last verified follow-up. No imputation of missing follow-up or covariate data was performed, and analyses were conducted using available cases. Survival probabilities were estimated using the Kaplan–Meier method. Pointwise 95% confidence intervals were calculated from Greenwood's standard errors and are displayed as a shaded band in Figure 3. In accordance with recommended practice for Kaplan–Meier reporting, the number of patients at risk at baseline and at each yearly follow-up landmark through 5 years is reported below the Kaplan–Meier curve in Figure 3 (13). Variables considered clinically relevant and/or associated with mortality in univariable analysis (*p* < 0.10) were entered into the multivariable Cox regression model. No *a priori* sample-size calculation was performed because the cohort comprised all eligible patients recorded in the registry during the predefined inclusion period. Given the limited cohort size and number of events, the Cox analyses were considered exploratory and hypothesis-generating and were not intended to establish validated independent predictors. No center-level adjustment or internal model validation was performed. Results are reported as hazard ratios and 95% confidence interval (95% CI) and *p*-values. A two-sided *p*-value <0.05 was considered statistically significant. Statistical analyses were performed using IBM SPSS Statistics version 25 (IBM Corp., Armonk, NY, USA).

## Results

### Study population and baseline characteristics

Of the 1,318 TAVI procedures recorded between January 2013 and December 2021, 68 were performed in patients aged ≥90 years. After eligibility assessment, eight patients were excluded, resulting in a final cohort of 60 patients (Figure 1). The distribution across participating centers was as follows: 25 patients (41.7%) were treated at Hesperia Hospital Modena, 14 (23.3%) at Ospedale Maggiore di Parma, 10 (16.6%) at Arcispedale Santa Maria Nuova, four (6.7%) at Ospedale Civile Sant’Agostino-Estense, four (6.7%) at Policlinico di Modena, and three (5.0%) at Ospedale Civile di Piacenza (Figure 2).

**Figure 1:**
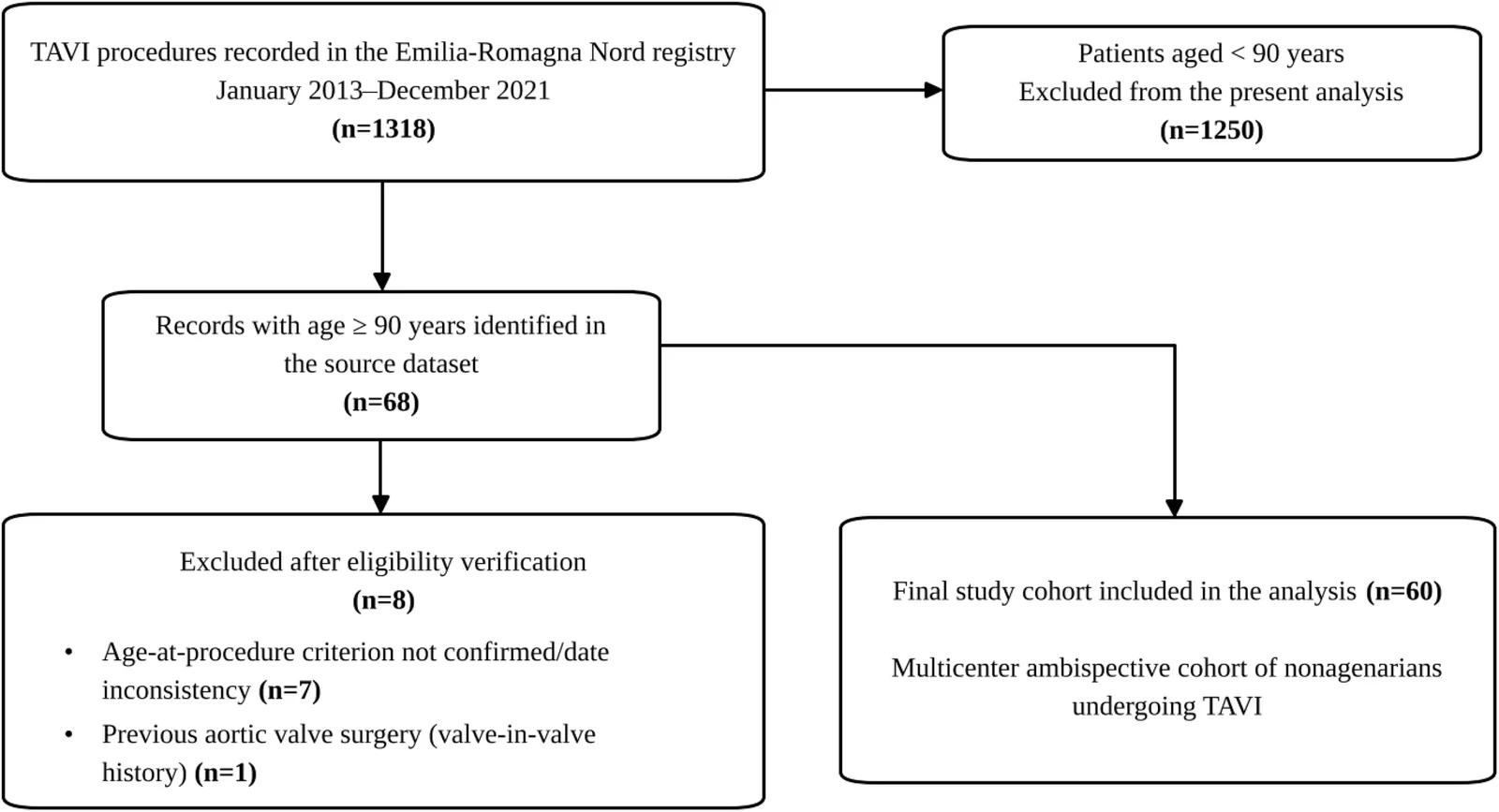
Flow chart of study population selection from the emilia-romagna north TAVI registry.

**Figure 2:**
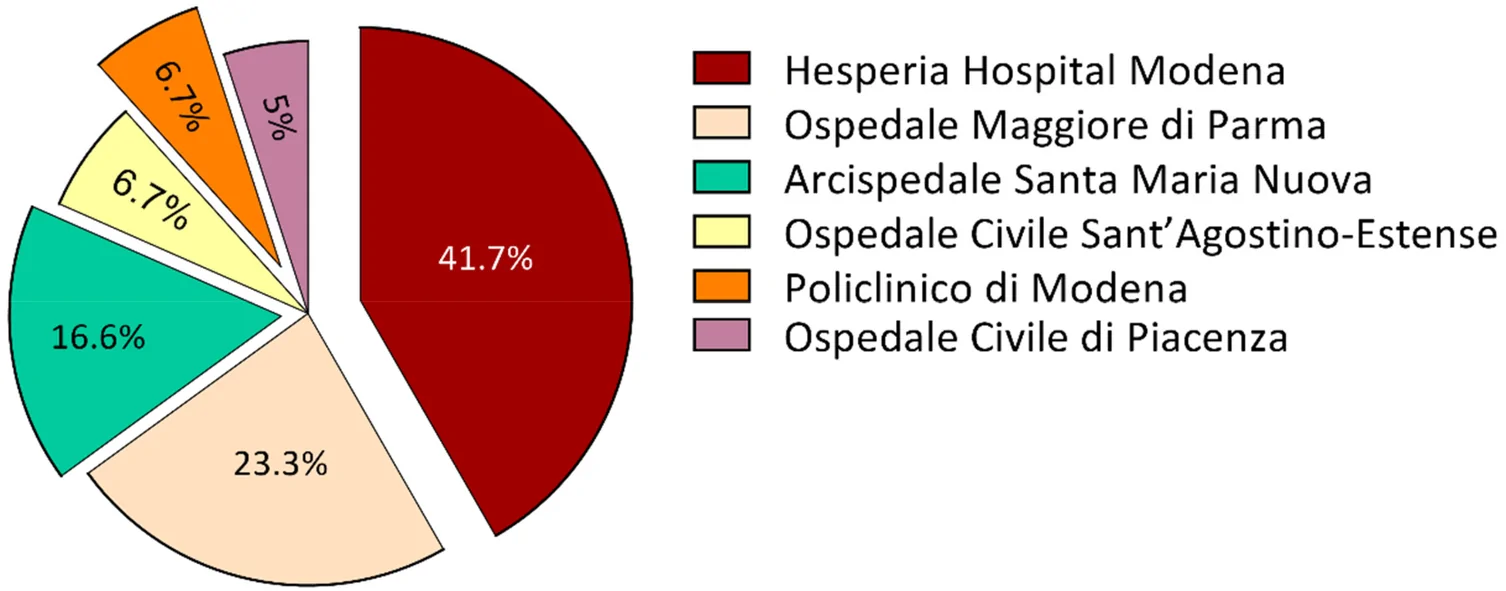
Distribution of the 60 nonagenarian patients across the six participating centers in the Emilia-Romagna North TAVI registry.

The mean age at the time of TAVI was 91 ± 1.5 years, and 40 patients (67%) were female. The mean observation time from TAVI to death or the last confirmed vital-status assessment was 5.7 ± 2.1 years, with a median of 5.0 years (IQR, 3.7–6.5). Baseline clinical characteristics are reported in Table 1.

**Table 1:** Baseline clinical and demographic characteristics of the study population, overall and according to vital status at follow-up.

		Total	Deceased	Alive at last follow-up	*P* value
Number, %		60	39 (65%)	21 (35%)	
Age, y		91 ± 1.5	91 ± 1.3	91 ± 1.6	0.5
BMI, kg/m^2^		25 ± 4	24 ± 4	25 ± 4	0.4
Sex	Female	40 (67%)	25 (64%)	15 (71%)	0.5
	Male	20 (33%)	14 (36%)	6 (29%)	
Comorbidities					
Hypertension, yes, %		56 (93%)	37 (95%)	19 (90%)	0.5
Diabetes, yes, %		7 (12%)	5 (13%)	2 (10%)	0.7
Dyslipidemia, yes, %		37 (62%)	26 (67%)	11 (52%)	0.3
Active smoker, yes, %		8 (13%)	5 (13%)	3 (14%)	0.8
COPD, yes, %		7 (12%)	7 (18%)	0	0.04
eGFR, mL/min/1.73m^2^		46 (35–68)	46 (38–63)	46 (33–72)	0.99
Previous Stroke, yes, %		5 (8%)	5 (13%)	0	0.09
CAD, yes, %		12 (20%)	7 (18%)	5 (24%)	0.6
AF, yes, %		16 (27%)	11 (28%)	5 (24%)	0.7
RBBB on ECG, yes, %		3 (5%)	0	3 (14%)	-
NYHA III-IV, yes, %		48 (80%)	29 (74%)	19 (90%)	0.19
STS score, %		5 ± 3	5 ± 2	6 ± 4	0.3
Observation time from TAVI to death or last vital-status assessment, y		5.7 ± 2.1			

Data presented as mean ± SD or Inter quartile range (IQR) and number and percentage.

Independent sample t-test or Mann–Whitney U test and Chi-Square test or Fisher's exact test has been done.

BMI, body mass index; COPD, chronic obstructive pulmonary disease; eGFR, estimated glomerular filtration rate; CAD, coronary artery disease; AF, atrial fibrillation; RBBB, right bundle branch block; NYHA, New York Heart Association; STS score, Society of Thoracic Surgeons score.

The prevalence of hypertension, diabetes, dyslipidemia, smoking, coronary artery disease, atrial fibrillation, and renal dysfunction did not differ significantly according to vital status at follow-up. COPD was present in seven of 39 deceased patients (18%) and in none of the 21 surviving patients (*p* = 0.04). Right bundle branch block was observed in none of the deceased patients and in three of the 21 surviving patients (14%). NYHA functional class III–IV was present in 29 of 39 deceased patients (74%) and 19 of 21 surviving patients (90%; *p* = 0.19). The mean STS score did not differ between deceased and surviving patients (5 ± 2% vs. 6 ± 4%; *p* = 0.3).

All patients had symptomatic severe aortic stenosis. The mean aortic valve area was 0.65 ± 0.2 cm^2^, the mean transaortic gradient was 51 ± 15 mmHg, and the mean STS score was 5 ± 3%. Left ventricular ejection fraction was ≤35% in 13 patients (22%). The mean transaortic gradient and aortic valve area did not differ significantly according to vital status. Moderate-to-severe aortic regurgitation was present in 17 of 39 deceased patients (44%) and eight of 21 surviving patients (38%; *p* = 0.6). Mitral regurgitation ≥2 + was present in 25 of 39 deceased patients (64%) and eight of 21 surviving patients (38%; *p* = 0.05) (Table 2).

**Table 2:** Preprocedural echocardiographic characteristics of the study population, overall and according to vital status at follow-up.

Echocardiography parameters	Total	Deceased (*n* = 39)	Alive at last follow-up (*n* = 21)	*P* value
LVEF, %	49 ± 11%	48 ± 10	49 ± 13	0.97
LVEF≤35%	13 (22%)	7 (18%)	6 (29%)	0.3
Mean gradient, mmHg	51 ± 15	49 ± 12	56 ± 20	0.1
AVA	0.65 ± 0.2	0.7 ± 0.2	0.6 ± 0.2	0.4
AR 3-4+	25 (42%)	17 (44%)	8 (38%)	0.6
MR ≥ 2+	33 (55%)	25 (64%)	8 (38%)	0.05

Data are presented as mean ± SD, median (IQR), or number (%), as appropriate. Comparisons were performed using the independent-samples t-test or Mann–Whitney U test for continuous variables and the chi-square test or Fisher's exact test for categorical variables, as appropriate.

LVEF, left ventricular ejection fraction; AVA, aortic valve area; AR, aortic regurgitation; MR, mitral regurgitation.

### Procedural characteristics and early outcomes

The remaining five procedures were performed through non-transfemoral access. However, the specific alternative access route was not consistently available in the harmonized multicenter registry and could therefore not be reported reliably. A balloon-expandable prosthesis was implanted in 32 patients (53%), and a self-expandable prosthesis was implanted in 28 patients (47%) (Table 3).

**Table 3:** Procedural characteristics, access route, prosthesis type, and periprocedural events in the study population, overall and according to vital status at follow-up.

	Total	Deceased (*n* = 39)	Alive at last follow-up (*n* = 21)	*P*-value
Self-expandable prosthesis, %	28 (47%)	19 (49%)	9 (43%)	0.7
Balloon-expandable prosthesis, %	32 (53%)	20 (51%)	12 (58%)	0.7
Transfemoral access, %	55 (92%)	35 (90%)	20 (95%)	0.5
AKI, %	10 (17%)	8 (21%)	2 (10%)	0.5
Contrast, mL	202 ± 101	219 ± 109	170 ± 74	0.07
Stroke/TIA/RIND within 30 days, %	1 (2%)	1 (3%)	0	0.99
Major bleeding (VARC2), %	9 (15%)	7 (18%)	2 (10%)	0.4
Moderate to severe AR post-procedure, %	6 (10%)	4 (10%)	2 (10%)	0.3
Pacemaker post-procedure, %	8 (13%)	5 (13%)	3 (14%)	0.99
Major vascular complication, %	6 (10%)	4 (10%)	2 (10%)	0.3
Cardiac tamponade, %	1 (2%)	1 (3%)	0	0.99

Data presented as mean ± SD or interquartile range (IQR) and number and percentage.

Independent sample t-test or Mann–Whitney U test and Chi-Square test or Fisher's exact test has been done.

TAVI, transcatheter aortic valve implantation; AKI, acute kidney injury; TIA, transient ischemic attack; RIND, reversible ischemic neurological deficit; AR, aortic regurgitation.

Study-defined procedural success was achieved in 58 of 60 procedures (97%). Two patients experienced major acute complications: one hemorrhagic shock secondary to a femoral vascular complication requiring urgent surgery and one cardiac tamponade caused by annular rupture requiring surgical conversion. Both patients survived the acute event.

The study-defined composite early safety endpoint at 30 days occurred in 20 patients (33.3%). Acute kidney injury occurred in 10 patients (16.7%), major bleeding in nine (15%), major vascular complications in six (10%), postprocedural pacemaker implantation in eight (13.3%), cerebrovascular events within 30 days in one (1.7%), and cardiac tamponade in one (1.7%). Moderate-to-severe postprocedural aortic regurgitation was documented in six patients (10%). No additional standardized postprocedural or longitudinal echocardiographic measurements were available for comparative analysis. None of the individual early safety components differed significantly according to vital status at follow-up (Table 3).

In-hospital mortality was 5% (three patients), including two deaths within 72 h due to cardiogenic shock and one death due to major stroke. Cumulative mortality at 30 days was 8.3% (five patients). No periprocedural myocardial infarctions were observed.

### Long-term survival

A date of death or last confirmed alive assessment was available for all 60 patients, and all patients therefore contributed to the time-to-event analysis. Thirty-nine deaths occurred, while 21 patients were right-censored at their last confirmed vital-status assessment. No event-status or survival-time data were missing for the primary endpoint. Nine deaths occurred within 1 year and 21 within 3 years, corresponding to crude observed survival proportions of 85% and 65%, respectively. The corresponding Kaplan–Meier estimated survival probabilities were 85% (95% CI, 76%–94%) at 1 year, 65% (95% CI, 53%–77%) at 3 years, and 35% (95% CI, 23%–47%) at 5 years. The numbers of patients at risk at baseline and at 1, 2, 3, 4, and 5 years were 60, 51, 47, 39, 30, and 21, respectively. The survival curve, the corresponding pointwise 95% confidence interval, and the annual risk-set counts are shown in Figure 3.

**Figure 3:**
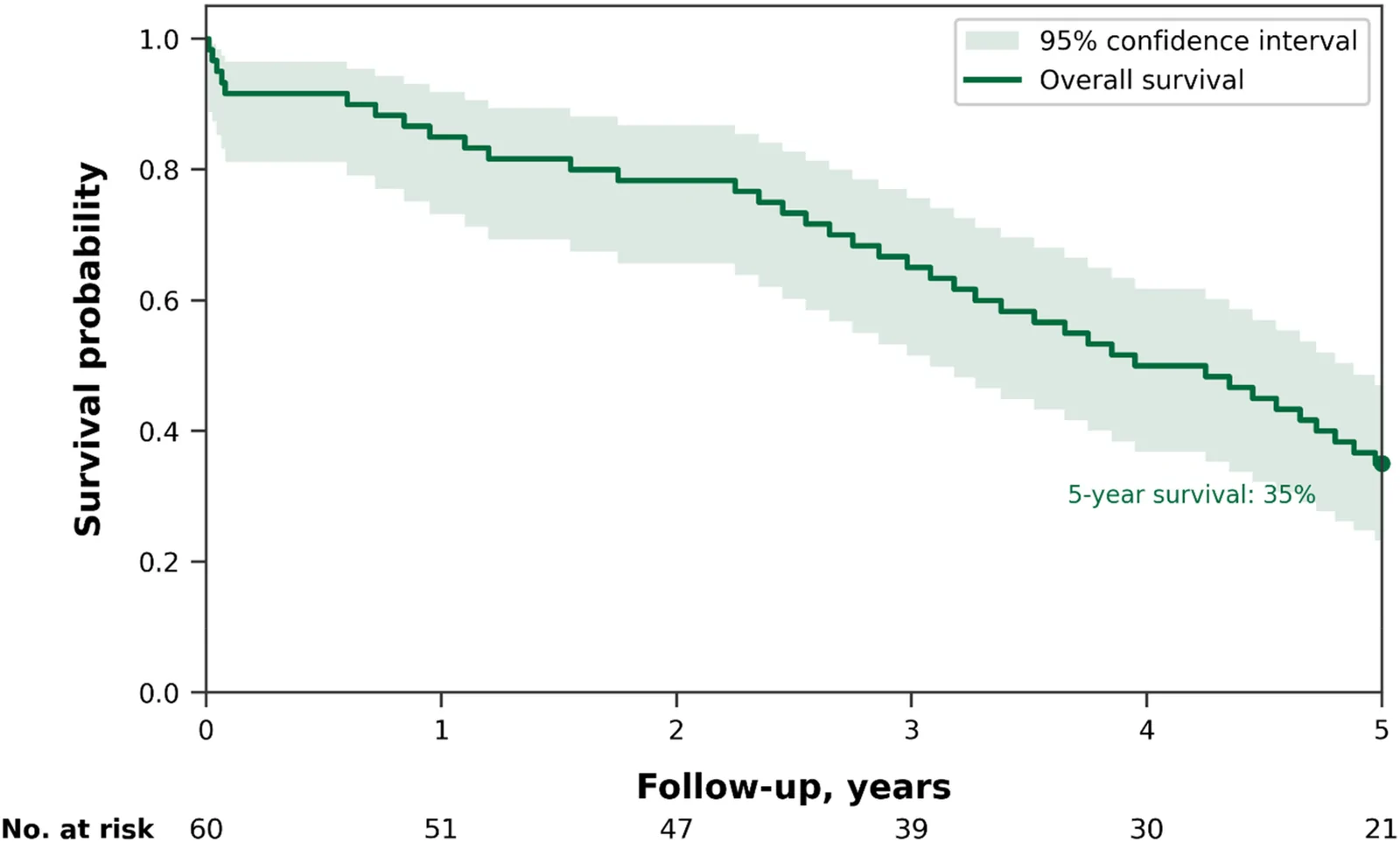
Kaplan–Meier curve of overall survival after TAVI in nonagenarian patients. The solid green line represents the Kaplan–Meier estimate, while the shaded area represents the pointwise 95% confidence interval calculated from Greenwood's standard errors. Follow-up is displayed up to the prespecified 5-year primary endpoint; estimated 5-year survival was 35%. The numbers of patients at risk at baseline and at each yearly follow-up time point are displayed below the *x*-axis.

### Exploratory Cox regression analyses

In the univariable Cox regression analysis, male sex was associated with mortality (HR, 2.0; 95% CI, 1.1–3.9; *p* = 0.03), as were COPD (HR, 3.2; 95% CI, 1.3–7.6; *p* = 0.006) and mitral regurgitation ≥2+ (HR, 2.0; 95% CI, 1.0–3.9; *p* = 0.03).

In the multivariable model, male sex was associated with mortality (HR, 2.5; 95% CI, 1.2–5.0; *p* = 0.02), as was COPD (HR, 2.9; 95% CI, 1.2–7.2; *p* = 0.02). Mitral regurgitation ≥2 + was not statistically significant in the multivariable model (HR, 1.9; 95% CI, 0.9–3.7; *p* = 0.08). LVEF ≤35% was not associated with mortality (HR, 0.8; 95% CI, 0.3–1.9) (Figure 4).

**Figure 4:**
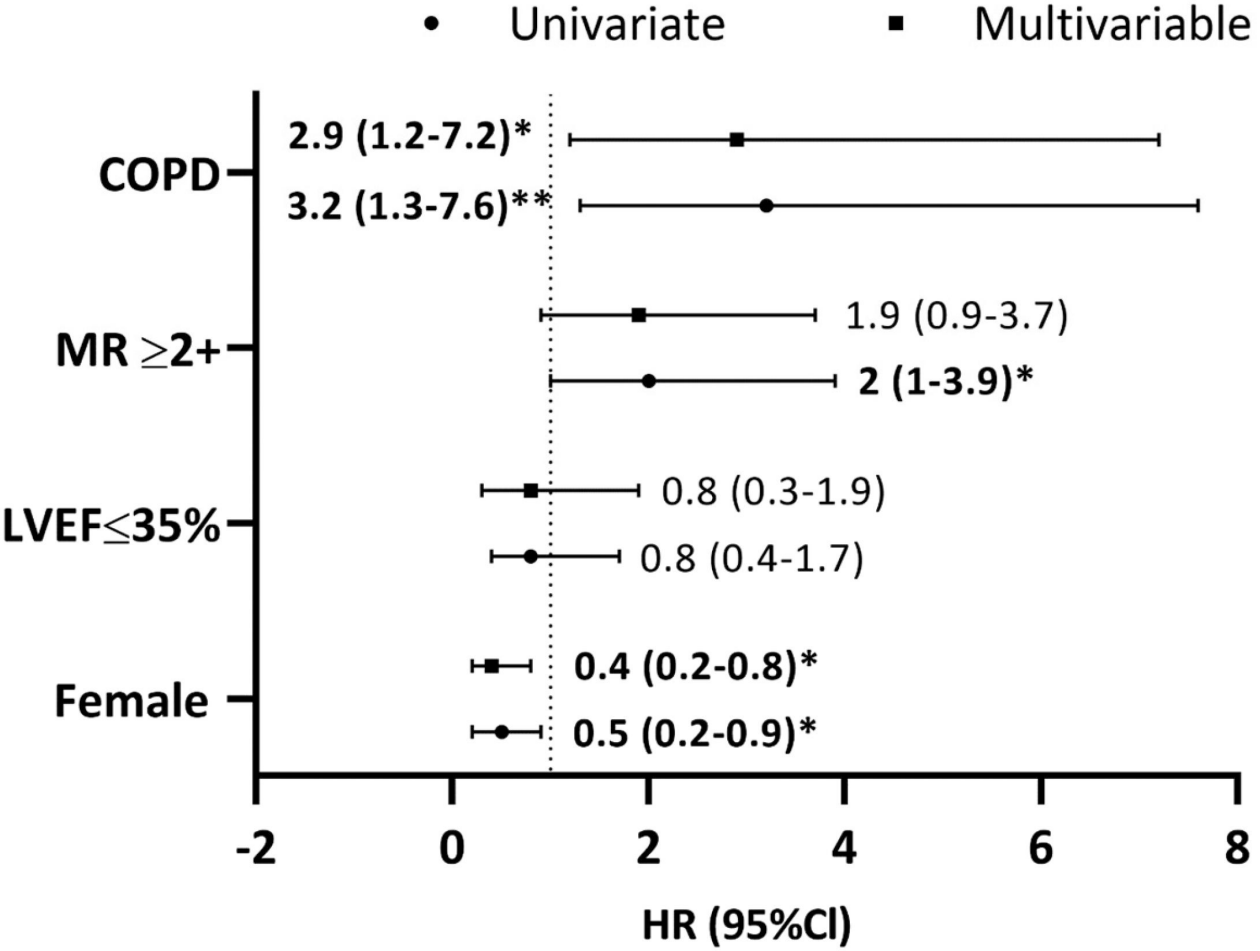
Univariable and multivariable cox regression analysis of selected clinical and echocardiographic variables associated with long-term mortality after TAVI. The multivariable model included all variables shown in the figure. Results are presented as hazard ratios (HR) with 95% confidence intervals (CI). *p < 0.05; **p < 0.01. Because of the limited sample size, these estimates are exploratory and should not be interpreted as validated prognostic predictors.

## Discussion

In this multicenter real-world study conducted across six centers in Emilia-Romagna and including 60 consecutive nonagenarian patients undergoing TAVI, the primary finding was a Kaplan–Meier estimated 5-year survival of 35%, despite a mean age of 91 years. This long-term outcome was accompanied by a procedural success rate of 97%, an in-hospital mortality of 5%, and a 30-day mortality of 8.3%. Therefore, describing the 5-year survival as merely ‘non-negligible’ would understate its clinical relevance: the estimate indicates that approximately one in three carefully selected nonagenarians was alive 5 years after treatment.

The early outcomes of our cohort were broadly consistent with those reported in the STS/ACC TVT and German Aortic Valve registries (4, 6). More importantly, the clinical relevance of the 35% 5-year survival estimate becomes clearer when considered against both background mortality and the poor prognosis of severe symptomatic aortic stenosis when definitive valve replacement is not performed. Barth et al. reported that, among carefully selected nonagenarians undergoing TAVR, mortality converged with that of an age- and sex-matched general population after 2 years, with an estimated 5-year survival of 40.8%. In a nonagenarian-specific comparative cohort, Bernard et al. also observed significantly better survival after TAVR than with medical therapy, together with survival comparable to that of an age- and sex-matched general population. Consistently, in the randomized PARTNER 1 trial of inoperable patients with severe symptomatic aortic stenosis, 5-year all-cause mortality was 71.8% after TAVR compared with 93.6% with standard treatment (HR, 0.50; 95% CI, 0.39–0.65; *p* < 0.0001). Although these populations are not directly interchangeable with our cohort, this external evidence indicates that the 35% 5-year survival observed in our study is clinically meaningful and supports a therapeutic benefit of TAVI in carefully selected nonagenarians (7, 14–16).

Taken together, these findings argue that chronological age alone should not be equated with therapeutic futility and support TAVI in appropriately selected nonagenarians; contemporary multicenter data also document favorable long-term survival and sustained functional improvement in this population. However, because our study did not include an untreated control group or perform a direct comparison with an age- and sex-matched population, the therapeutic benefit remains an inference supported by external comparative evidence rather than a causal effect demonstrated by the present study. Selection bias and the small number of patients remaining at risk at 5 years must also be considered when interpreting the absolute survival estimate (2, 17).

The age distribution of the cohort requires specific consideration. Although eligibility included all patients aged ≥90 years, the mean age of 91 ± 1.5 years indicates that the study population was concentrated at the younger end of the nonagenarian spectrum. This pattern likely reflects referral and Heart Team selection processes, as patients approaching the upper end of the 90–99-year age range may have been less likely to be evaluated or considered suitable for intervention, particularly in the presence of advanced frailty or major comorbidities. Consequently, our findings principally describe carefully selected, relatively younger nonagenarians and should not be extrapolated without caution to patients aged 95 years or older. The observed outcomes may therefore be more favorable than those expected in an unselected nonagenarian population.

In the exploratory Cox model, male sex and COPD were associated with higher long-term mortality, whereas at least moderate mitral regurgitation showed a signal in preliminary analyses but did not retain statistical significance in the final model.

The descriptive analyses were consistent with this interpretation, as COPD was present exclusively in the deceased group and at least moderate mitral regurgitation was more frequent among non-survivors. However, these observations should be regarded as purely descriptive and do not support robust prognostic inferences, particularly in light of the small size of some subgroups.

These findings are consistent with the existing literature, in which female sex has been associated with better long-term survival after TAVI beyond one year, a difference attributed to factors including smaller body surface area, lower prevalence of coronary artery disease, and more favourable left ventricular remodelling, while COPD identifies patients with a greater burden of frailty, reduced functional reserve, and higher long-term mortality (18–21).

Additional support comes from the prospective three-center Japanese study by Yokoyama et al., which enrolled 767 patients undergoing TAVI, including 94 nonagenarians. Three-year survival did not differ significantly between patients aged ≥90 and those aged <90 years (77.9% vs. 82.5%; *p* = 0.25), and age ≥90 years was not independently associated with mortality. Instead, female sex, body mass index, NYHA functional class, and renal function were associated with long-term outcome (22, 23). These findings further support assessment of biological reserve and clinical status rather than chronological age alone (24).

From a practical perspective, these findings reinforce the concept that selection of nonagenarian candidates for TAVI should not be based solely on chronological age or traditional surgical risk scores. In this age group, a structured assessment of frailty, functional status, and biological reserve is likely to be more relevant for estimating the true potential for benefit. The literature has shown that frailty is associated with increased risk of death and disability after aortic valve replacement, and that summary tools such as the Essential Frailty Toolset have important prognostic value. Accordingly, current guidelines recommend that treatment decisions be made in a patient-centered manner by a multidisciplinary Heart Team, with geriatric expertise involved when appropriate (11, 25). These findings highlight the importance of individualized decision-making beyond chronological age.

The heterogeneous nature of follow-up must also be considered when interpreting these findings. Patients who had died before prospective contact contributed time-to-event information through retrospectively ascertained dates of death but could not contribute prospective functional assessments. Among patients contacted prospectively, outpatient visits and telephone contacts were not supported by a uniform protocol for serial assessment of NYHA functional class, frailty, exercise capacity, independence in activities of daily living, or quality of life. Consequently, the study can describe survival but cannot determine whether longer survival was accompanied by preserved functional independence, symptom improvement, or satisfactory quality of life. This represents a major limitation in nonagenarians, for whom patient-centered functional outcomes may be as important as mortality. “Future prospective studies should also assess participation in and adherence to individualized cardiac rehabilitation, given its potential relevance to functional capacity and quality of life and the substantial age- and comorbidity-related barriers to participation” (26, 27). All-cause mortality provides a consistently ascertainable measure of overall prognosis but does not distinguish cardiovascular deaths from competing mortality related to frailty, pre-existing comorbidities, or other age-related conditions. Because causes of long-term death were not systematically adjudicated, cardiovascular and non-cardiovascular mortality could not be analyzed without a substantial risk of misclassification. Therefore, the survival findings should not be interpreted as a direct measure of TAVI-specific benefit. The absence of statistically significant associations between individual early safety events and long-term mortality suggests that, in nonagenarians, late prognosis may depend more on baseline clinical vulnerability than on individual periprocedural events, although it should be acknowledged that the meta-analysis by Noguchi et al. (2022) showed that, compared with younger patients, nonagenarians had increased early and late mortality, major or life-threatening bleeding, stroke, and major vascular complications (10, 28).

Overall, our findings suggest that TAVI should not be ruled out *a priori* in nonagenarian patients, but rather considered within a multidimensional selection process aimed at identifying those with a reasonable expectation of clinical, functional, and prognostic benefit. In clinical practice, these results support a selective approach to TAVI in nonagenarians, prioritizing biological rather than chronological age.

### Limitations

The findings of the present study should be interpreted in light of several limitations. First, this was an ambispective observational cohort study conducted in a relatively small population, with the inherent limitations of a non-randomized design, which does not allow definitive conclusions or causal inferences. Second, the absence of a conservatively treated control group or an alternative treatment strategy precludes assessment of the relative benefit of TAVI compared with other therapeutic options in nonagenarian patients. In addition, the study population consisted of patients carefully selected by the Heart Team, resulting in potential selection bias. The narrow age distribution (mean age, 91 ± 1.5 years) indicates that patients at the younger end of the nonagenarian spectrum were overrepresented; therefore, the cohort cannot be considered representative of the entire 90–99-year age range. Referral and treatment-selection processes may have contributed to more favorable outcomes and substantially limit the generalizability of the findings, particularly to patients aged 95–99 years, those with advanced frailty or major comorbidities, and those who were not referred for TAVI. Patient contribution across centers was also uneven, with a substantial proportion of the cohort contributed by a limited number of centers, which may have introduced center-related bias related to referral patterns, patient selection, procedural experience, and local practice. In addition, the registry did not capture a harmonized baseline assessment of frailty, functional status, cognition, nutritional status, activities of daily living, or estimated life expectancy, precluding verification that candidate-selection criteria were applied consistently across centers. This unmeasured heterogeneity may have materially influenced both cohort composition and the observed outcomes and should be regarded as a major source of selection bias. Further heterogeneity may have derived from both the multicenter design and the long enrolment period, during which selection criteria, implanted devices, and clinical practice may have changed over time. Future multicenter studies should adopt standardized serial echocardiographic protocols, including a multiparametric assessment of right ventricular function; recent inter-operator data suggest that TDI S’ may provide greater reproducibility than TAPSE (29, 30). Although a multivariable analysis was performed, the limited sample size and the relatively low number of events per variable may have resulted in model overfitting. Therefore, the results of the Cox regression analysis should be interpreted with caution and considered exploratory rather than confirmatory. The COPD estimate was affected by quasi-complete separation, as COPD was present in seven patients, all of whom died, and in none of the surviving patients. Similarly, the difference observed for right bundle branch block was based on only three cases and should not be interpreted as evidence of a protective association. Furthermore, the small number of patients remaining at risk at 5 years resulted in substantial uncertainty surrounding the 5-year Kaplan–Meier estimate. Another major limitation is the absence of standardized longitudinal functional assessment. For patients who had already died before prospective contact, outcome ascertainment was necessarily limited to vital status and date of death. Moreover, prospective contacts with surviving patients did not uniformly include validated measures of frailty, NYHA functional class, exercise capacity, independence in activities of daily living, or quality of life. Therefore, the study cannot establish whether survival after TAVI was accompanied by sustained functional or quality-of-life benefit. Complete and independently adjudicated information on the causes of long-term death was not systematically available. Accordingly, cardiovascular and non-cardiovascular mortality could not be analyzed reliably, and the use of all-cause mortality does not distinguish deaths potentially related to aortic stenosis or TAVI from competing mortality due to frailty, comorbidities, or other age-related conditions.

## Conclusion

In this ambispective multicenter observational cohort study, TAVI in nonagenarian patients carefully selected by the Heart Team was associated with a high procedural success rate and a clinically meaningful Kaplan–Meier estimated 5-year survival of 35%. However, the present study evaluated survival and procedural outcomes only and cannot determine the effect of TAVI on functional status, independence, or quality of life. In our cohort, male sex and the presence of COPD showed an exploratory association with higher mortality during follow-up. Overall, these findings suggest that chronological age alone should not represent the sole criterion for decision-making and support a selective approach to TAVI in carefully selected patients, predominantly at the younger end of the nonagenarian age range. Extrapolation of these results to older and more frail nonagenarians requires caution.

## Data Availability

The raw data supporting the conclusions of this article will be made available by the authors, without undue reservation.

## References

[1] Depalo D Giordano C. Demography and the current account: a case-study of Italy. Banca D’Italia. (2025). 10.2139/ssrn.6202999

[2] Galatas C Afilalo J. Transcatheter aortic valve replacement over age 90: risks vs benefits. Clin Cardiol. (2020) 43:156–62. 10.1002/clc.23310 31840834 PMC7021650

[3] O’Sullivan CJ Wenaweser P. Optimizing clinical outcomes of transcatheter aortic valve implantation patients with comorbidities. Expert Rev Cardiovasc Ther. (2015) 13:1419–32. 10.1586/14779072.2015.1102056 26479904

[4] Blumenstein J Möllmann H Bleiziffer S Bauer T Ensminger S Bekeredjian R. Transcatheter aortic valve implantation in nonagenarians: insights from the German aortic valve registry (GARY). Clin Res Cardiol Off J Ger Card Soc. (2020) 109:1099–106. 10.1007/s00392-020-01601-4 31989251

[5] Majid M Rosenzveig A Haroun E Arockiam AD Agrawal A Sorathia S. A contemporary review of transcatheter aortic valve replacement in low-gradient aortic stenosis. Brain Heart. (2024) 3:4017. 10.36922/bh.4017

[6] Arsalan M Szerlip M Vemulapalli S Holper EM Arnold SV Li Z. Should transcatheter aortic valve replacement be performed in nonagenarians?: insights from the STS/ACC TVT registry. J Am Coll Cardiol. (2016) 67:1387–95. 10.1016/j.jacc.2016.01.055 27012397 PMC5443624

[7] Mack MC Szerlip M Herbert MA Akram S Worley C Kim RJ. Outcomes of treatment of nonagenarians with severe aortic stenosis. Ann Thorac Surg. (2015) 100:74–80. 10.1016/j.athoracsur.2015.02.045 26013708

[8] Khalili H Bansal P Al Taii H Morcos R Kucharik M Mathews A. Quality of life outcomes after transcatheter aortic valve replacement in nonagenarians. J Invasive Cardiol. (2020) 32:375–9. 10.25270/jic/20.00027 32999091

[9] Güney MC Bozkurt E. Transcatheter aortic valve implantation in nonagenarians: a comparative analysis of baseline characteristics and 1-year outcomes. J Cardiovasc Dev Dis. (2025) 12:327. 10.3390/jcdd12090327 PMC12470840 41002606

[10] Noguchi M Ueyama H Ando T Takagi H Toshiki K. Clinical outcomes in nonagenarians undergoing transcatheter aortic valve implantation: a systematic review and meta-analysis. Cardiovasc Interv Ther. (2022) 37:202–8. 10.1007/s12928-021-00755-w 33428156

[11] Vahanian A Beyersdorf F Praz F Milojevic M Baldus S Bauersachs J. 2021 ESC/EACTS Guidelines for the management of valvular heart disease. Eur Heart J. (2022) 43:561–632. 10.1093/eurheartj/ehab395 34453165

[12] Von Elm E Altman DG Egger M Pocock SJ Gøtzsche PC Vandenbroucke JP. Strengthening the reporting of observational studies in epidemiology (STROBE) statement: guidelines for reporting observational studies. Br Med J. (2007) 335:806–8. 10.1136/bmj.39335.541782.AD PMC2034723 17947786

[13] Morris TP Jarvis CI Cragg W Phillips PPJ Choodari-Oskooei B Sydes MR. Proposals on Kaplan–Meier plots in medical research and a survey of stakeholder views: kMunicate. BMJ Open. (2019) 9:e030215. 10.1136/bmjopen-2019-030215 31575572 PMC6773317

[14] Barth S Hautmann MB Reents W Zacher M Griese DP Kerber S. Transcatheter aortic valve replacement for severe aortic stenosis can improve long-term survival of nonagenarians as compared to an age- and sex-matched general population. J Cardiol. (2020) 75:134–9. 10.1016/j.jjcc.2019.07.014 31474498

[15] Bernard J St-Louis R Robichaud M Kalavrouziotis D Dumont É Paradis J-M. Is transcatheter aortic valve replacement futile in patients over ninety years of age? CJC Open. (2023) 5:784–91. 10.1016/j.cjco.2023.08.001 37876887 PMC10591129

[16] Kapadia SR Leon MB Makkar RR Tuzcu EM Svensson LG Kodali S. 5-year outcomes of transcatheter aortic valve replacement compared with standard treatment for patients with inoperable aortic stenosis (PARTNER 1): a randomised controlled trial. Lancet. (2015) 385:2485–91. 10.1016/S0140-6736(15)60290-2 25788231

[17] Madanat L Allam M Khalili H Rabah A Tariq R Zamzam M. Long-term survival and quality of life following transcatheter aortic valve replacement in nonagenarians. Am J Cardiol. (2024) 213:140–5. 10.1016/j.amjcard.2023.12.031 38134979

[18] Conrotto F D’Ascenzo F Presbitero P Humphries KH Webb JG O’Connor SA. Effect of gender after transcatheter aortic valve implantation: a meta-analysis. Ann Thorac Surg. (2015) 99:809–16. 10.1016/j.athoracsur.2014.09.089 25633460

[19] Dziewierz A Tokarek T Kleczynski P Sorysz D Bagienski M Rzeszutko L. Impact of chronic obstructive pulmonary disease and frailty on long-term outcomes and quality of life after transcatheter aortic valve implantation. Aging Clin Exp Res. (2018) 30:1033–40. 10.1007/s40520-017-0864-y 29185204 PMC6096867

[20] Coppi F Cavalletti A Pagnoni G Campani C Grossule F Maini A. Pulmonary hypertension in patients with sjögren’s syndrome: gender differences in cardiovascular risk factors and instrumental data. Int J Cardiol. (2025) 428:133131. 10.1016/j.ijcard.2025.133131 40058609

[21] Coppi F Pagnoni G Grossule F Nassar A Maini A Masaracchia G. Gender-specific differences in diastolic dysfunction and HFpEF: pathophysiology, diagnosis, and therapeutic strategies. J Cardiovasc Dev Dis. (2025) 12:213. 10.3390/jcdd12060213 40558648 PMC12194537

[22] Yokoyama H Tobaru T Muto Y Hagiya K Higuchi R Saji M. Long-term outcomes in Japanese nonagenarians undergoing transcatheter aortic valve implantation: a multi-center analysis. Clin Cardiol. (2019) 42:605–11. 10.1002/clc.23183 30989700 PMC6553359

[23] Bao M-H Lv Q-L Li H-G Zhang Y-W Xu B-F He B-S. A novel putative role of TNK1 in atherosclerotic inflammation implicating the Tyk2/STAT1 pathway. Mediators Inflamm. (2020) 2020:1–9. 10.1155/2020/6268514 PMC7368939 32694928

[24] Savonitto G Barbisan D Ameri P Lombardi CM Driussi M Gentile P. Characteristics, prognosis, and European society of cardiology and European respiratory society risk stratification in patients with obesity and pulmonary arterial hypertension. Chest. (2025) 168:749–62. 10.1016/j.chest.2025.04.008 40250800

[25] Afilalo J Lauck S Kim DH Lefèvre T Piazza N Lachapelle K. Frailty in older adults undergoing aortic valve replacement: the FRAILTY-AVR study. J Am Coll Cardiol. (2017) 70:689–700. 10.1016/j.jacc.2017.06.024 28693934

[26] Pagnoni G Vicenzi A Darroudi S Maini A Sbarra F Marangi F. Adherence to exercise and functional rehabilitation programs in patients with cardiovascular diseases: barriers and strategies. J Cardiovasc Dev Dis. (2025) 13:8. 10.3390/jcdd13010008 41590835 PMC12842067

[27] Coppi F Pagnoni G Vicenzi A Darroudi S Savino G Zurlo C. Long-term attrition in a 12-month adapted physical activity program after cardiac rehabilitation: a real-world longitudinal study. J Cardiovasc Dev Dis. (2026) 13:418. 10.3390/jcdd13090418 42783025 PMC13607313

[28] Zeng Q Cai H Yuan X Jiang F Li H Dong X. Lymphoplasmapheresis for steroid-refractory neuromyelitis optica spectrum disorder: a real-world multicenter study in China. CNS Neurosci Ther. (2025) 31:e70628. 10.1111/cns.70628 41074269 PMC12513854

[29] Pagnoni G Giuggioli D Darroudi S De Pinto M Maini A Spinella A. Reproducibility of echocardiographic measurements of right ventricular function: comparison between TAPSE and TDI s’. J Cardiovasc Dev Dis. (2026) 13:104. 10.3390/jcdd13030104 41892693 PMC13026364

[30] de Pinto M Coppi F Spinella A Pagnoni G Morgante V Macripò P. The predictive role of the TAPSE/sPAP ratio for cardiovascular events and mortality in systemic sclerosis with pulmonary hypertension. Front Cardiovasc Med. (2024) 11:1430903. 10.3389/fcvm.2024.1430903 39469124 PMC11513352

